# Waived Consent in Perinatal/Neonatal Research—When Is It Appropriate?

**DOI:** 10.3389/fped.2019.00493

**Published:** 2019-11-26

**Authors:** Wade D. Rich, Anup C. Katheria

**Affiliations:** Neonatal Research Institute, Sharp Mary Birch Hospital for Women & Newborns, San Diego, CA, United States

**Keywords:** informed consent, resuscitation, neonalal, waiver, ethics

## Abstract

Informed consent is a process ensuring that subjects enrolled in research are appropriately informed of the risks and benefits. While this process is well-defined when it is possible and practical to obtain consent prior to the research intervention, it can be less clear in cases of deferred or waived consent. Defining minimal risk, such as when research is attempting to determine which of two currently practiced interventions is safest and/or most effective, is critical to moving forward in establishing appropriate care in newborns. For perinatal/neonatal research the challenge lies between the ethical justification for approaching women in labor or under medication vs. the scientific integrity of excluding a number of subjects that may potentially benefit the most from an intervention. Researchers must work with their IRBs as well as families who have participated in trials to determine the most appropriate method for obtaining informed consent from expectant parents. Clinical researchers and IRBs ultimately need to find a middle ground for the appropriate use of deferred or waived consent.

## Introduction

Informed consent is a process ensuring that subjects enrolled in research, or in the case of newborns their parents or guardians, are appropriately informed of the risks and benefits of the research. The consent process is straightforward when it can be obtained directly from the parents or guardians prior to the intervention. When the timing or emergency nature of the intervention suggests it is not practical to obtain consent beforehand, other options may be considered.

For the studies done at or near the time of birth approaching the parents or guardians prior to the intervention requires antenatal consent. The timing of antenatal consent may affect the generalizability of such trials, and there are practical and ethical issues surrounding approaching pregnant women in labor or under medication. In a retrospective review of six Neonatal Research Network trials that all included prospective consent Foglia et al. (1) found no significant difference in primary or secondary outcomes when comparing enrolled subjects with eligible but not enrolled subjects, but found significance in the need for CPR. All subjects in the review were prospectively consented, and the majority of non-enrolled were due to parent refusal of consent. In a previous study (2) of the effects of antenatal consent during a multicenter randomized controlled trial of neonatal resuscitation interventions in very preterm infants (3) the authors found that women enrolled in the trial were significantly more likely to be have been insured, had prenatal care, and been given antenatal steroids than those who were eligible but not approached, and their infants were significantly less likely to die and/or have severe intraventricular hemorrhage/periventricular hemorrhage (IVH/PVL). Songstad et al. (4) performed a secondary analysis of a trial comparing nasal high flow (nHF) with nasal continuous positive airway pressure (CPAP) for primary respiratory support in preterm infants (5, 6). Because it included periods of retrospective and prospective consent there was a unique opportunity to compare the two methods. The researchers found that mothers enrolled in the retrospective cohort were less likely to have been given a full course of antenatal steroids and to have received antibiotics. These studies suggest that prospective consent for trials that occur at or near delivery increase the risk of enrolling a non-representative population; specifically, not including the sickest infants who might benefit most from the intervention.

The ethics of the antenatal approach include questions of whether women in labor are able to remember what was asked of them (7, 8) whether they have the capacity to make an informed decision (9), and whether it is even ethical to approach them for consent (10). It is difficult to quantify whether remembering what was asked is an issue of pregnancy/labor. Ballard et al. interviewed women who participated in a trial of morphine in newborns found that while 95% of women who understood the reason for the study could remember benefits of the trial, only 2 could state the risks, but also showed a lack of recall in fathers (11).

When informed consent cannot be obtained before delivery another option is to wait until the procedure/intervention has already occurred (12). In the United States, informed consent can only occur prior to enrollment, and “deferred” consent is not an acceptable term. Thus, only the waiver process is available as an alternative. While some studies in the states have chosen to approach parents after the intervention, for instance to continue to collect data, this is not seen by US regulators as a *deferred* consent, but rather a waiver of consent for the primary intervention, followed by an informed consent for the collection of data. In a 1993 letter to Institutional Officials and IRB Chairs, the Office for Protection from Research Risks, now Office for Human Research Protections (OHRP) stated that “informed consent procedures which provide for other than legally effective and *prospectively obtained* consent, fail to constitute informed consent under the Health and Human Services regulations for the protection of human research subjects” (13).

Waiver has been used in neonatal trials where the interventions being studied are deemed to be minimal risk and obtaining antenatal consent prior to the intervention is not possible/practicle (14–16). These two conditions, that the research is minimal risk, and that it could not practicably be carried out without the requested waiver or alteration, are spelled out in the Code of Federal Regulations 45 CFR § 46.116(f) [previously 116(d)]. These two requirements are at the heart of the question as to whether it is reasonable and appropriate to do research under a waiver of consent.

## Minimal Risk

Under current US regulations, the first and most decisive component to accept a trial for waiver of consent is the requirement that the trial be “no more than minimal risk.” In the common rule 45 CFR § 46.102(j) states: “*Minimal risk means that the probability and magnitude of harm or discomfort anticipated in the research are not greater in and of themselves than those ordinarily encountered in daily life or during the performance of routine physical or psychological examinations or tests.”* In trials that occur in vulnerable populations, such as preterm infants, this definition is problematic because all preterm infants are at greater risk than what is ordinarily encountered in daily life for a term infant. Some researchers believe that comparative effectiveness trials that meet the other regulatory requirements for waiver should be allowed when two interventions are minimal risk. The problem, as brought to light in the discussions of the SUPPORT trial, is how one defines and ultimately presents the risks of a trial. SUPPORT was a 2 × 2 factorial trial comparing two types of early respiratory support (nasal CPAP vs. endotracheal intubation with surfactant) and two ranges of arterial oxygen saturations (85–89 and 90–95 percent). Informed consent was obtained prior to birth (17). The trial found no difference the primary outcome, but found increased death in infants randomized to the lower oxygen range, but questions arose about why the increased risk of death was not disclosed to the families in the trial. Subsequently, OHRP wrote a draft guidance, “Disclosing Reasonably Foreseeable Risks in Research Evaluating Standards of Care,” stating that, in comparing two *standard of care* treatments, research risks need to be disclosed if “each individual subject will face potentially different risks than he or she might face without enrollment” (13). As noted by Lantos in a response to this draft (18), the common rule delineates risks that are already part of the subjects daily care from those *added* by the research, stating that IRBs considering the risk of a trial should focus on those risks and benefits which are directly related to the research, not those that the subject would receive whether they were in the trial or not [Code of Federal Regulations 45 CFR 46, Section 46.111a(2)]. Trials have been conducted to study the question of whether being in a trial, as suggested by OHRP, is inherently a risk. In a Cochrane review including 86,640 patients treated in randomized controlled trials Vist et al. (19) compared equivalent subjects receiving interventions during research trials with those receiving the same intervention clinically. They concluded that trial participation did not affect trial outcomes when compared to the same outcomes of non-participants. Fernandes et al. included studies from two different groups (20). They included trials in which a treatment was under research guidelines and the same treatment a part of clinical treatment, as well as cases in which treatment was only available within a research trial. In both cases, they were able to find no evidence to suggest that trial participants were better or worse off than non-participants. In 9 studies within the subgroup where an effective intervention was only available to participants, those not participating in the trial had significantly worse health outcomes (mean difference −0.36, 95% CI −0.61 to −0.12).

Lantos used a theoretical trial of intubation for meconium aspiration syndrome to query leading researchers, clinicians, and bioethicists on whether they felt it was appropriate to waive consent (21). Two groups found that because intubation and suctioning were part of the standard of care, the trial was no more than minimal risk, while another felt that the risks of laryngoscopy and intubation should be considered and that the trial could not be waived. This issue of whether risks that are added by participating in the trial should be considered by an Institutional Review Board when determining minimal risk and by extension the ability to approve a waiver of consent has yet to be resolved.

## Feasibility

In a recommendation published in October of 2016 the Secretary's Advisory Committee on Human Research Protections (SACHRP), a committee of the Office of Human Research Protection under the department of Health and Human Services, stated that practicality can be defined using either the standard of administrative feasibility or scientific validity (22). They suggest that a trial may claim that obtaining consent is administratively infeasible if there are too many subjects, or a large number that are difficult to contact due to loss to follow-up or representing a unique population. However, a large number of subjects in and of itself is not a valid reason for waiver if it is possible to interact with all subjects in the trial. It is, according to these recommendations, reasonable to state that a consent is not *scientifically* feasible when a significant number of subjects cannot be approached, such as would be the case when a number of mothers in a trial of neonatal resuscitation are admitted emergently and cannot be consented.

## Cluster Randomized Trials

One possible way to eliminate the problem of decreased generalizability caused by the process of antenatal consent is the use of a cluster randomized trial design. Conceptually this design requires a waiver of consent, because the cluster, not the individual, is the unit of randomization. For example, a current multicenter Cluster Randomized Trial randomizes each center to one of two cord clamping interventions, with a crossover at the halfway point of the trial (NCT03631940). If either of the interventions is of greater than minimal risk, this type of trial cannot be practically conducted in the US due to the requirement for individual consent. The Secretary's Advisory Committee on Human Research Protections (SACHRP) has stated that it recommends allowing for individual assessment of risk for the two interventions, so that if there is a “control” or placebo group, only the intervention that has greater than minimal risk would require consent (23). The rationale is if you study two interventions, and one of those interventions has the possibility of being “better” than the other, at least one group of subjects is then at greater than minimal risk. Now the overall study is considered greater than minimal risk and is no longer eligible for a waiver of consent, and SACHRP has suggested that it is acceptable for IRBs to make independent risk determinations for individual arms in a cluster trial. In other words, it should be acceptable under current regulations that one arm might involve minimal risk to subjects and therefore not require consent, while another cluster in the same protocol may be judged to involve more than minimal risk to subjects (22).

## Parental Reaction to Waived or Deferred Consent

One important concern regarding trials that have a waived/deferred consent is how parents will react when they learn the intervention was done without prior informed consent. In a trial conducted in 14 hospitals throughout England and Wales, children requiring emergency admission were randomized to one of 3 central line catheters. The families of children admitted for an emergency provided deferred consent at a higher rate than those admitted on an elective basis [84% v 69 (*n* = 9 sites)]. Rich et al. (24) conducted a survey of how parents whose infants participated in the Umbilical Cord Milking vs. Delayed Cord Clamping in Preterm Infants (PREMOD) trial felt about how the waiver of consent process effected their baby's care (25, 26). Amongst those who participated in the survey, all had positive or neutral feelings about the effect of the waiver, and no parents had a negative response. The timing of this survey is unique in that the parents were participants in a waivered consent process. Though the survey did not ask specifics, all subjects responded that they remembered being approached and consented for a trial. In a trial by Ayers et al. of antenatal consent for similar umbilical cord related interventions, responses were positive, but many parents did not remember having been enrolled, and felt that approaching them outside of the stressful delivery period would be helpful (27).

The question of how parents feel about waived or deferred consent has not been fully answered likely due to differing populations. Studies that have included only parents that have experienced a high-risk pregnancy and delivery show positive responses about deferred consent compared to the general population. For example, Culbert et al. surveyed 318 well-educated parents with good prenatal care. Of the 102 who responded the researchers found that parents preferred conventional pre-intervention consent to delayed or waived consent (28). Surveys conducted by Burgess et al. in both prospective and retrospective cases found that fewer than 10% of parents were comfortable with physicians making the decision to enroll their baby in a trial (29). It is important to differentiate between the attitudes of parents with a neonatal intensive care unit experience with those who have not. Our group has developed a parent advisory board consisting of mothers and fathers of former preterm infants some of which have participated in trials. Their input has allowed us to determine the best approach for trial design, consent language and a member sits on the ethics review board during review of neonatal trials. We believe that any institution considering using waivered consent develop such a board as part of their research operations.

### Emergency Trials

When obtaining informed consent prior to the intervention in perinatal/neonatal trials is either not practical or scientifically invalid, but the trial is not determined to be minimal risk, waiver of consent is allowed under 45 CFR 46.101 (i). The requirements for obtaining the waiver include that the situation is life threatening, no proven better treatment option exists, and the data is needed to show safety and effectiveness.

As with questions of minimal risk and feasibility, the question of whether a premature newborn is in a life-threatening situation is not always clear. Most trials of neonatal resuscitation/transition involve incremental improvements in care, not life or death decisions. Truog et al. described several situations where interventions done under the rubric of research that required the same intervention which was done as part of clinical care (30). They suggest that the requirement for consent in a clinical trial be based on specific criteria involving availability of the treatment, the level of risk, and whether the subject might have a preference between treatments.

## Conclusion

Conducting clinical trials during the perinatal period can have many issues and challenges. Researchers must work with their IRBs as well as families who have participated in trials to determine the most appropriate method for obtaining informed consent from expectant parents. While antenatal consent is ideal, there may be some instances where a more ethical approach may actually be waiver or deferred consent. All of these determinations depend on appropriate definitions for minimal risk, what risks should be included in the informed consent, and whether interventions can be considered emergent. These critical pathways will allow for a broader scope of future neonatal research.

## Author Contributions

WR drafted the initial manuscript and revisions and approved final manuscript to be submitted. AK provided substantial assistance in the revision and final draft of the manuscript.

### Conflict of Interest

The authors declare that the research was conducted in the absence of any commercial or financial relationships that could be construed as a potential conflict of interest.
